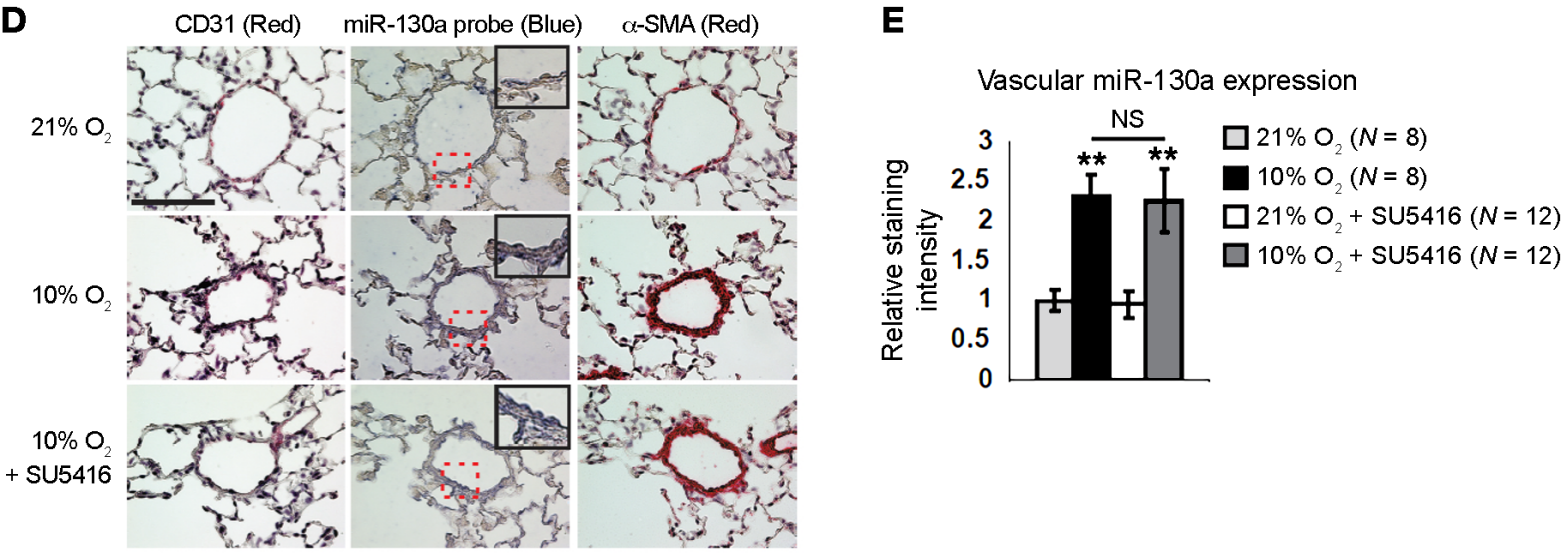# Systems-level regulation of microRNA networks by miR-130/301 promotes pulmonary hypertension

**DOI:** 10.1172/JCI161077

**Published:** 2022-05-16

**Authors:** Thomas Bertero, Yu Lu, Sofia Annis, Andrew Hale, Balkrishen Bhat, Rajan Saggar, Rajeev Saggar, W. Dean Wallace, David J. Ross, Sara O. Vargas, Brian B. Graham, Rahul Kumar, Stephen M. Black, Sohrab Fratz, Jeffrey R. Fineman, James D. West, Kathleen J. Haley, Aaron B. Waxman, B. Nelson Chau, Katherine A. Cottrill, Stephen Y. Chan

Original citation: *J Clin Invest*. 2014;124(8):3514–3528. https://doi.org/10.1172/JCI74773

Citation for this corrigendum: *J Clin Invest*. 2022;132(10):e161077. https://doi.org/10.1172/JCI161077

The authors recently became aware that in Figure 3D, lung images from a 10% O_2_–exposed mouse were inadvertently presented in the row labeled as lung images from a 10% O_2_ + SU5416–exposed mouse. The authors reviewed the original data and provided the correct version of Figure 3D with data obtained from a replicate experiment. In addition, the key for Figure 3E was missing information. The correct versions of Figure 3, D and E appear below. The authors have stated that these errors did not affect signal quantification, interpretations, or conclusions of the study.

The authors regret these errors.

## Figures and Tables

**Figure:**